# Selective DNA encapsulation in extracellular vesicles of *Saccharomyces cerevisiae*

**DOI:** 10.20517/evcna.2025.118

**Published:** 2026-03-31

**Authors:** Ana Perea-Martínez, Miguel Mejías-Ortiz, Pilar Morales, Ramon Gonzalez

**Affiliations:** Instituto de Ciencias de la Vid y del Vino (CSIC, Universidad de La Rioja, Gobierno de La Rioja), Logroño 26007, Spain.

**Keywords:** Extracellular vesicles, plasmid DNA, *Saccharomyces cerevisiae*, DNA packaging, DNA delivery

## Abstract

**Aim:** Extracellular vesicles (EVs) are emerging mediators of intercellular communication capable of transporting nucleic acids, including plasmid and genomic DNA. This study aimed to investigate the presence, enrichment, and protection of distinct DNA classes in EV-enriched fractions from *Saccharomyces cerevisiae* (*S. cerevisiae*).

**Methods:** Two plasmids were analyzed: the high-copy YEp352 (2µ) and the centromeric pRS316. EV-enriched fractions were isolated from yeast culture supernatants, and the presence of plasmid, ribosomal [18S ribosomal DNA (RDN18)], and mitochondrial [cytochrome c oxidase subunit I (COX1)] DNA was assessed. DNase digestion assays were performed to evaluate DNA protection, and transformation assays were conducted using *S. cerevisiae* and *Escherichia coli*. Enrichment of endogenous *vs.* exogenously added plasmid DNA was also compared.

**Results:** Both plasmids were strongly enriched in EV fractions relative to culture supernatants. However, only pRS316 exhibited partial resistance to DNase digestion, suggesting encapsulation, while YEp352 remained fully susceptible. Despite this protection, pRS316-associated EVs did not mediate transformation of yeast or bacterial recipients, indicating compromised integrity or inefficient delivery. Endogenously produced plasmid DNA showed significantly higher enrichment than exogenously added DNA. Ribosomal and mitochondrial DNA were also detectable in EVs but were highly sensitive to DNase degradation, indicating minimal protection.

**Conclusion:** These findings demonstrate that selective DNA incorporation into EVs depends on both DNA type and intrinsic plasmid features. The results suggest that plasmid properties influence EV-mediated transport and protection, highlighting the selective and cargo-specific nature of DNA packaging in yeast EVs.

## INTRODUCTION

Extracellular vesicles (EVs) have emerged as key mediators of intercellular communication, capable of transporting a diverse array of biomolecules, including proteins, lipids, and nucleic acids, across a wide range of organisms^[1,2]^. These vesicles are particles released by the cells that are delimited by a lipid bilayer, and display a considerable heterogeneity in size, ranging from 20 to 500 nanometers in diameter^[3]^.

EVs are involved in several biological processes. For instance, the role of these structures in fungi has attracted increasing attention due to their contribution to physiological processes such as cell wall remodeling, antifungal resistance, pathogenesis, stress responses and microbial interactions^[4-7]^. Indeed, EVs isolated from fungi have been established as potent mediators of intraspecies communication. This is the case of EVs from *Paracoccidioides brasiliensis* and *Aspergillus fumigatus*, which act as stress response signals within cells of the same species. Additionally, the same authors demonstrated that EVs from *Candida albicans *(*C. albicans*) can promote the yeast-to-hyphal transition^[8]^. Interestingly, interspecific effects have also been reported. EVs from *Saccharomyces cerevisiae* (*S. cerevisiae*) and *Histoplasma capsulatum* can inhibit hyphal differentiation in *C. albicans*, according to some studies^[9]^. Also in fungi, it has been reported that EVs are capable of enhancing thermotolerance and drug resistance^[10,11]^.

EVs produced by fungi display a complex composition, encompassing proteins of diverse function (metabolism, stress response, translation, virulence), RNA, lipids, carbohydrates and pigments^[12]^. Remarkably, most of the proteins identified in fungal EVs lack classical signal peptides that direct proteins to the endoplasmic reticulum, indicating incorporation via unconventional secretion pathways^[13]^. While no specific markers have been established for EVs in* S. cerevisiae, *studies in* C. albicans *have identified promising putative markers, including the plasma membrane proteins Sur7 and extracellular vesicle protein 1 (orf19.6741) (Evp1). These proteins may represent fungal equivalents of the tetraspanins commonly used to define mammalian exosomes^[14]^. EVs must traverse the rigid yeast cell wall to reach the extracellular space, and proteomic analyses have revealed the presence of numerous cell wall-degrading enzymes within these vesicles, suggesting that enzymatic hydrolysis of wall components may facilitate their passage^[13]^. Furthermore, current evidence suggests that the formation of yeast EVs is a complex process that involves multiple cellular pathways, including post-Golgi secretion, multivesicular body-related mechanisms, GRASP (Golgi ReAssembly Stacking Proteins) and autophagy-related processes^[15]^.

Focusing on the industrial context, yeast communication via EVs appears to play a relevant role. For example, in winemaking, EVs have been identified as pivotal elements in mediating microbial interactions. In this context, recent evidence indicates that EVs secreted by wine yeast strains are capable of modulating *S. cerevisiae* transcriptomic responses in a similar manner to whole cells^[16]^, supporting their role in yeast communication.

Some studies in prokaryotes, specifically gram-negative bacteria, have reported the entrapment of DNA in a specific type of EV known as outer-inner membrane vesicles (OIMVs), although the underlying mechanisms remain unclear^[17]^. More broadly, DNA transport via EVs in both prokaryotes and lower eukaryotes appears to contribute to horizontal gene transfer and host immune response^[18-20]^. In more complex organisms such as mammalian systems, EV-associated DNA is gaining increasing attention due to its potential roles in cancer progression and therapy resistance^[21,22]^.

In fungi, RNA molecules have been identified within EVs^[23-25]^ and some studies have highlighted the role of proteins and RNA from the EVs in fungal virulence^[26]^. Specifically in *S. cerevisiae*, EVs have been shown to deliver both microRNAs and large messenger RNAs (mRNAs) to a variety of recipient cells^[27]^.

While RNA trafficking has received increasing attention, the mechanisms governing DNA packaging, protection, and delivery via EVs in *S. cerevisiae* remain largely unknown. In environments where different microbial species coexist, transport of DNA through EVs may represent a novel mechanism of horizontal gene transfer. If DNA contained in EVs is taken up and functionally integrated by recipient cells, this process could have implications in the evolution of microbial communities, contributing to genetic diversification and adaptation. In this context, model systems such as wine fermentation, where diverse yeasts and bacteria interact closely, could offer an experimentally accessible framework.

In this study, we analyze EV-enriched fractions from *S. cerevisiae* to assess the presence and protection of different DNA types, including chromosomal and extrachromosomal elements. We focus primarily on plasmids of varying copy number to determine whether this characteristic influences their packaging. Our findings provide new insights into DNA transport by yeast EVs and contribute to a better understanding of extracellular genetic dynamics in yeast.

## METHODS

### Strains and culture media

*S. cerevisiae *BY4741 (*MATa, his3Δ1, leu2Δ0, met15Δ0, ura3Δ0*) and BY4743 (*MATa/MATα his3Δ1/his3Δ1 leu2Δ0/leu2Δ0 met15Δ0/MET15 LYS2/lys2Δ0 ura3Δ0/ura3Δ0*) strains were used in this study. 

Minimal medium (MM) contained 1.7 g/L yeast nitrogen base (YNB) without amino acids and without ammonium sulphate, 5 g/L ammonium sulphate, 20 g/L glucose and 20 g/L agar if required.

BY4743 strain was grown in MM supplemented with uridine 20 mg/mL, leucine 60 mg/L and histidine 20 mg/L.

BY4741 cells were transformed with YEp352 or pRS316 plasmids and selected in MM plates supplemented with leucine 60 mg/L, histidine 20 mg/L and methionine 20 mg/L, but not supplemented with uridine, as prototrophy is provided by the plasmids.

*Escherichia coli *(*E. coli*) DH5α cultures were grown in lysogeny Broth (LB) ampicillin (10 g/L NaCl, 10 g/L tryptone, 5 g/L yeast extract, 100 mg/L ampicillin, pH 7.0) or LB ampicillin plates (LB supplemented with 20 g/L agar and 100 mg/L ampicillin) at 37 °C.

*E. coli* DH5α competent cells were chemically transformed following the instructions of the manufacturer (Invitrogen, Carlsbad, CA, USA) and transformants were selected on LB ampicillin plates.

Pre-cultures of transformed yeast were grown in 20 mL MM with the needed amino acids but lacking uridine, and cultures were done in Yeast Peptone Dextrose Broth (YPD) (20 g/L glucose, 20 g/L peptone and 10 g/L yeast extract).

### Plasmid types

The plasmids YEp352 (5,181 bp; 2µ-based, high-copy) and pRS316 [4,887 bp; Chromosome VI centromere/Autonomously Replicating Sequence 4 (CEN6/ARSH4)-based, low-copy] were used in this study. Both are yeast-*E. coli* shuttle vectors carrying the URA3 selectable marker and an ampicillin resistance gene for propagation in *E. coli*.

### Yeast transformation

The transformation of BY4741 strain with plasmids YEp352 and pRS316 was validated by polymerase chain reaction (PCR) amplification of plasmid-specific replication origin sequences: 2µ for YEp352 and Chromosome centromere/Autonomously Replicating Sequence (CEN/ARS) for pRS316 [Supplementary Table 1]. PCR products were analyzed by electrophoresis on a 1% agarose gel to verify the presence of the expected bands.

### Isolation of EV-enriched fractions from BY4741

EV-enriched fractions from BY4741-YEp352 and BY4741-pRS316 strains were isolated following protocols from previous studies with some modifications^[6,16]^.

Forty-eight hours pre-cultures of these strains were washed twice to remove any medium and used to inoculate cultures in 350 mL MM media without uridine at an initial optical density at 600 nm (OD_600_) of 0.2. The culture was grown in 1-L flasks loosely sealed with aluminum foil to allow proper aeration and incubated at 25 °C with agitation at 150 revolutions per minute (rpm) for 48 h. 

Following incubation, cells were removed by centrifugation at 4,000 *×g* for 15 min. The resulting supernatant was supplemented with one tablet per litre of protease inhibitor [Complete Mini, ethylenediaminetetraacetic acid (EDTA)-free; Roche, Basel, Switzerland] and filtered through a 0.45 µm Nalgene Rapid-flow filtration device (ThermoFisher Scientific, Dreieich, Germany).

Concentration of the sample was performed at 4 °C using a tangential flow filtration unit MidiKros® hollow fiber filter module (100 kDa, Repligen, Waltham, MA; catalog number D04-E100-05-N) at a flow rate of approximately 200 mL/min and a transmembrane pressure of 1.2 bar. Filtration was carried out until the system reached its dead volume. At this point, three rounds of diafiltration were performed using 50 mL of phosphate-buffered saline (PBS) (137 mM NaCl, 2.7 mM KCl, 10 mM Na_2_HPO_4_, 2 mM KH_2_PO_4_, pH 7.4), each followed by ultrafiltration to the dead volume.

To recover EVs, dead volume was collected from the filter and additional 50 mL of PBS were passed through the filter unit. This wash was also concentrated to dead volume and recovered volume was combined with the previous one to yield a total of ~ 25 mL.

The concentrate was then centrifuged at 15,000 *×g* for 30 min at 4 °C to eliminate residual debris.

Afterwards, ultracentrifugation was conducted using 32 mL PC thick-walled tubes (25 mm × 89 mm; Beckman Coulter, Brea, CA, USA) at 100,000 *×g* (45,000 rpm) for 70 min at 4 °C, using a Beckman ultracentrifuge equipped with a 70Ti fixed-angle rotor.

Pellets were washed once with PBS, subjected to a second round of ultracentrifugation under the same conditions, and finally resuspended in 175 µL of PBS or DNase buffer to reach a 2,000-fold concentration of the initial culture volume.

All samples were used immediately or treated with DNase depending on the following experiment.

Fractions of every stage of the EVs isolation protocol were obtained and frozen at -20 °C.

Additionally, to verify plasmid retention during yeast cultivation, a dilution of the culture used for EV isolation was first grown on YPD plates. Colonies from these YPD plates were then replica plated onto uridine-free selective medium, and on average ~ 90% of colonies were able to grow, indicating that the majority of cells retained the plasmid.

### DNase treatment of EV-enriched fraction

A 100 µL aliquot of EVs in DNase buffer was incubated with DNase I (ThermoFisher Scientific, Dreieich, Germany) at 37 °C for 30 min to allow digestion of free DNA. DNase was subsequently inactivated by incubation at 70 °C for 15 min.

As a positive control for DNase activity, a dilution of plasmid DNA in DNase buffer was prepared and subjected to the same treatment, including incubation with DNase I and heat inactivation. This dilution was chosen to approximate the expected plasmid concentration (in this case, 170 pg/mL) present in the concentrated EV fraction.

Two negative controls were included: (1) plasmid DNA at the same concentration in DNase buffer without DNase I, and (2) EV samples resuspended in DNase buffer without DNase I. Both negative controls underwent the same thermal treatment as the enzyme-treated samples.

All samples were subsequently analyzed by quantitative PCR (qPCR) using primers specific for plasmid sequences, targeting the ampicillin resistance gene (*ampR*), to assess the presence of residual non-digested DNA.

### DNase protection assay with membrane disruption

EV-enriched pellets from the BY4741-pRS316 strain obtained by ultracentrifugation and resuspended in DNase buffer were used for the assay. Membrane disruption was performed by adding 0.5% Igepal CA-630 (Sigma) and incubating for 30 min at 4 °C, followed by DNase treatment as described above. Four conditions were prepared: untreated EVs, EVs treated with DNase, EVs treated with Igepal, and EVs treated with Igepal followed by DNase. Prior to these experiments, naked plasmid DNA was incubated with and without the detergent before DNase treatment to confirm that the detergent did not inhibit DNase activity.

### Assessment of exogenous plasmid DNA recovery

To compare the recovery of exogenously added plasmid DNA during EVs isolation *vs.* the endogenously produced plasmid, an appropriate dilution of naked plasmid pRS316 in YPD medium was used. This dilution was selected based on prior qPCR experiments demonstrating detectable amplification at this concentration, and corresponded to 17 pg/mL of naked plasmid.

Two experimental conditions were established. First, plasmid DNA was added directly to YPD medium before undergoing tangential flow filtration (naked). Second, a culture of BY4741 cells was grown for 48 h in YPD, after which the culture supernatant was centrifuged and filtered through 0.45 µm filters to completely remove cells as described above (naked + cells). Following filtration, plasmid DNA was added to the cell-free supernatant. This condition was performed to discard a potential association of plasmid DNA with cell-derived materials.

Samples of every fraction of the EVs isolation process were collected for posterior analysis by qPCR to assess the presence of free plasmid DNA.

### Quantitative PCR analysis

Specific primer pairs were designed by using Primer3 (version 4.1.0) software and used to amplify the ampicillin cassette of the plasmids pRS316 and YEp352, as well as 18S ribosomal DNA (*RDN18*) and cytochrome c oxidase subunit I (*COX1*) genes in each fraction [Supplementary Table 1]. qPCR was performed following the manufacturer’s instructions, with the SYBR**^TM^** Green PowerUp**^TM^** Master Mix (Applied Biosystems). Briefly, each 20 µL reaction included 10 µL of the master Mix, 1 µL of each primer (10 µM), 1µL of the sample and molecular biology water (Sigma) to complete the reaction. All reactions were performed in triplicate. Standard curves for the primer pair for each plasmid were generated with tenfold serial dilutions of plasmid DNA to determine primer efficiency. qPCR was performed in an Applied Biosystems**^TM^** 7500 Real-Time PCR System (Thermo Fisher Scientific, Waltham, MA, USA) with the following cycling conditions: UDG activation at 50 °C for 2 min, polymerase activation at 95 °C for 2 min, followed by 40 cycles of denaturation at 95 °C for 15 s, annealing at 55 °C for 30 s, and extension at 72 °C for 30 s. Cycle threshold (Ct) values were obtained in the same software. Relative DNA abundance was calculated from Ct values and expressed in fold change. For some analyses, fold change values were further expressed as percentages relative to a reference sample. In other experiments, data were presented as log_2_ fold change (log_2_FC) to facilitate comparison between groups. All calculations were performed in Microsoft Excel (Office LTSC Professional Plus 2021).

### EV-mediated plasmid transfer

To assess the potential for plasmid transfer via EVs in *E. coli*, EVs from BY4741-pRS316 were isolated using the described protocol, with the initial culture volume doubled to scale up EV production. EVs were isolated using standard differential centrifugation protocols and resuspended in PBS.

As a recipient, *E. coli* was cultured in LB medium at 37 °C with shaking. On the following day, cultures were diluted to an OD_600_ of 0.05 in fresh LB and incubated for 1-2 h at 37 °C (180 rpm) to reach early logarithmic phase (OD_600_ ~ 0.2-0.4) prior to exposure to EVs.

Three experimental conditions were applied to 4 mL aliquots of early-log *E. coli* cultures: addition of 175 µL of either EVs, free pRS316 plasmid (1:10^6^ dilution in PBS), or PBS as a negative control. All cultures were incubated at 37 °C for 18-19 h. Following incubation, each culture was plated on LB agar supplemented with ampicillin (100 µg/mL). Plates were incubated at 37 °C overnight.

The remaining cultures were transferred to LB-ampicillin medium and incubated at 37 °C with shaking. OD_600_ was measured to assess bacterial growth.

To assess the DNA uptake in yeast, the *S. cerevisiae* strain BY4743 was used as the recipient. Precultures were grown in MM supplemented with the appropriate amino acids and uridine at 25 °C for 48 h. On the following day, cells were harvested, washed twice with sterile water, and adjusted to an optical density of 4. A 100 µL aliquot of an EV-enriched fraction obtained from BY4741 transformed with pRS316 was added to the cell suspension. The mixture was incubated overnight at 25 °C in MM supplemented with the amino acids but lacking uridine.

The next day, cells were diluted to an OD of 0.2 and growth was monitored over the course of one week at 25 °C using a Hach 2100N turbidimeter. Additionally, selection for uridine was performed on agar plates lacking uridine. As with the *E. coli* experiments, comparable controls were included to assess the specificity and potential mechanism of transfer.

### Nanoparticle tracking analysis

Vesicle size distribution and concentration of the EV-enriched fractions were determined at the Center for Biomedical Research of La Rioja (CIBIR) using a NanoSight NS300 (Malvern Instruments Ltd.) equipped with Nanoparticle Tracking Analysis (NTA) software version 3.4 (Build 3.4.4). Samples were diluted 1:500 in PBS. Videos of 60 s were recorded at 25 frames per second and approximately 24 °C using a camera level of 11 and a detection threshold of 2.

### Statistical analysis

Experiments were performed in triplicate in three independent experiments, and the data are presented as mean ± standard deviation. Statistical comparisons between groups were performed using Student’s *t*-test. All analyses were performed using Microsoft Excel, Office LTSC Professional Plus 2021. Differences were considered significant at a level of *P* < 0.05.

## RESULTS

### Presence of plasmid DNA in the EV-enriched fraction

To investigate the presence of plasmid DNA in the EV-enriched fraction, two types of plasmids were used: YEp352, a high-copy-number 2µ plasmid, and pRS316, a centromeric plasmid. While YEp352 is characterized by its high copy number, pRS316 ensures a more stable distribution between mother and daughter cells during yeast division due to the presence of CEN/ARS sequences, albeit with a lower copy number.

**Figure 1:**
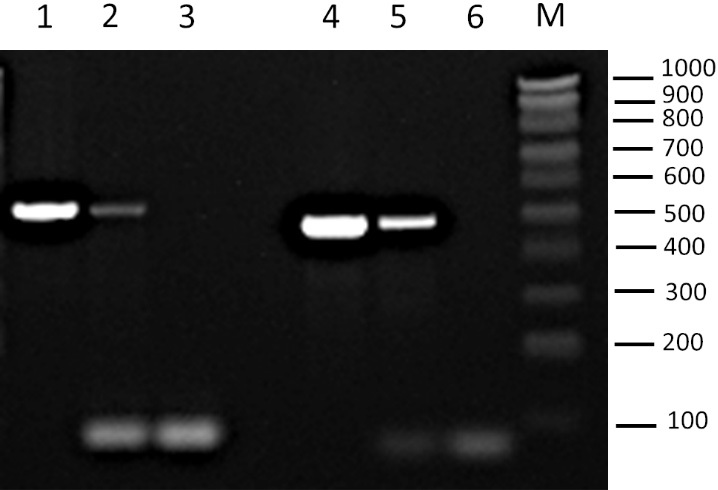
Detection of plasmids YEp352 and pRS316 by PCR amplification. Agarose gel showing PCR products from amplification of specific plasmid fragments in the EV-enriched fractions. Lane 1: YEp352 plasmid. Lane 2: EV fraction from BY4741-YEp352 strain; Lane 3: negative control (no template) for YEp352 primers; Lane 4: pRS316 plasmid. Lane 5: EV fraction from BY4741-pRS316 strain; Lane 6: negative control for pRS316 primers; M: Molecular weight marker NZYDNA Ladder V (100-1,000 bp). The amplified region corresponds to the 2μ origin for YEp352 and to the CEN/ARS sequence for pRS316. Positions of molecular weight markers are indicated on the right (bp). PCR: Polymerase chain reaction; EV: extracellular vesicle.

PCR analyses of the EV-enriched fractions consistently detected plasmid DNA for both pRS316 and YEp352 plasmids, indicating the presence of plasmid DNA in this fraction [Figure 1]. At this stage, it was not possible to determine whether the plasmid DNA was encapsulated within the vesicles or merely associated with them.

**Figure 2:**
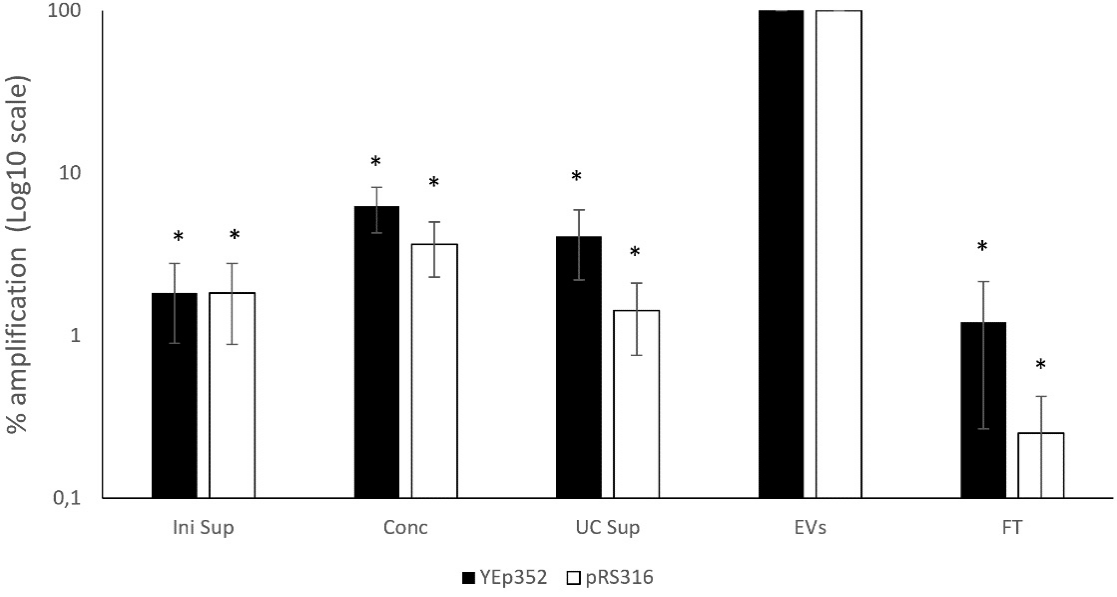
Relative abundance of amplified plasmid DNA across different fractions of the EVs isolation process for BY4741-YEp352 and BY4741-pRS316. Data are normalized to the EV-enriched fraction (set as 100%) and expressed as a percentage relative to the EV fraction. Data represent the mean ± standard deviation from three independent experiments. Significant differences *vs.* EV-enriched fraction of both plasmids were determined by using the Student’s *t*-test (*, *P* < 0.05). Levels of plasmids in the EV-enriched fractions were significantly higher compared to all fractions. For YEp352, significant differences were observed for Ini sup, Conc, UC Sup, and FT. For pRS316, significant differences were observed for Ini sup, Conc, UC Sup, and FT. FT: Flowthrough; Conc: concentrated sample after tangential flow filtration; UC Sup: ultracentrifugation supernatant; EV: extracellular vesicle.

To evaluate plasmid DNA enrichment during EV isolation, samples from different stages of the process were analyzed by qPCR, including the culture supernatant and intermediate fractions. When the percentage of amplified plasmid DNA was normalized relative to the EV-enriched fraction, the EV fraction showed the highest concentration for both plasmids [Figure 2]. Setting the EV-enriched fraction as 100%, the plasmid DNA level in the initial culture supernatant was below 2%. The remaining fractions contained significantly lower levels, ranging from nearly 0% to 6.2% for YEp352 and 3.7% for pRS316. These results demonstrate that both plasmids were strongly enriched in the EV fraction. Nanoparticle tracking analysis results of vesicle fractions from ultracentrifuge showed a mean particle diameter of 180.6 ± 13.4 nm and an estimated concentration of 8.32 ± 0.55 × 10^9^ particles/mL.

### Impact of DNase treatment on plasmid DNA integrity in EVs

To explore the protection of plasmid DNA within EVs, DNase treatment was applied to degrade free DNA, assuming that the DNA packaged in EVs would be protected from the action of DNase, unlike free DNA. To evaluate the extent of DNA degradation depending on plasmid concentration and DNase activity, we simultaneously performed qPCR on free plasmid samples at concentrations similar to those found in the EV-enriched fractions, both treated and untreated with DNase.

**Figure 3:**
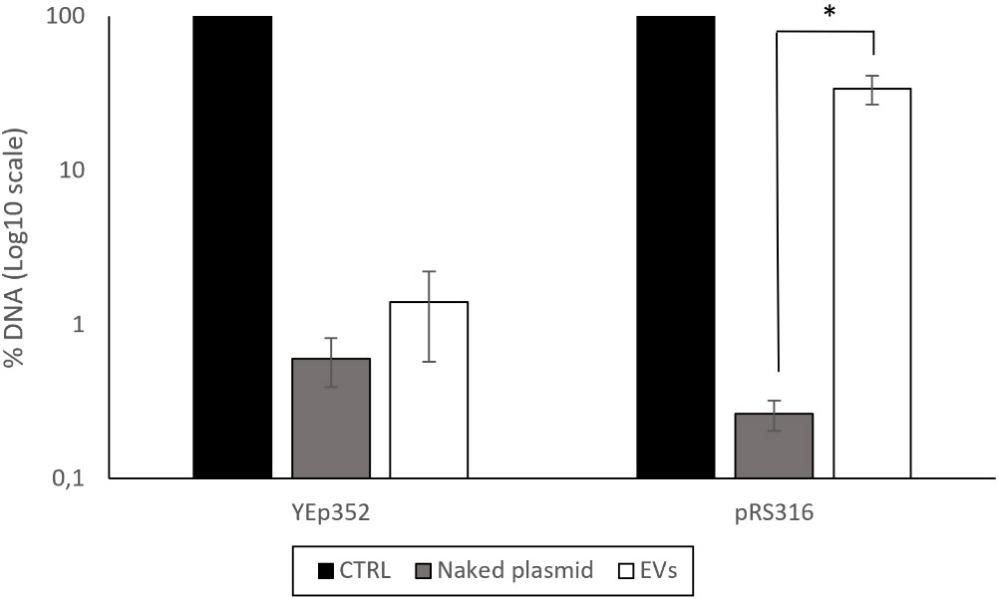
Percentage of DNA remaining after DNase treatment in the EV-enriched fractions isolated from BY4741-YEp352 and BY4741-pRS316. The non-treated sample, shown as the control and set to 100%, was used as the reference for data normalization. As an additional control for DNase activity, naked plasmids were treated at concentrations comparable to those present in the corresponding EV-enriched fractions. Data represent the mean ± standard deviation of three independent experiments. Statistical significance between the DNase-treated EV-enriched fraction and the naked plasmid condition for pRS316 was determined using a Student’s *t*-test (*, *P* < 0.05). CTRL: Control; EV: extracellular vesicle.

The results varied depending on the plasmid used. For the YEp352 plasmid, DNase treatment resulted in almost complete degradation of detectable plasmid DNA in the EV fraction [Figure 3]. This suggests that, despite its high concentration in the fraction, YEp352 DNA was not protected within EVs and remained accessible to DNase.

In contrast, the pRS316 plasmid exhibited partial protection following DNase treatment. Approximately 34% of the DNA remained detectable after the action of the enzyme. In comparison, only about 0.3% of the naked plasmid DNA was detected after DNase treatment [Figure 3].

**Figure 4:**
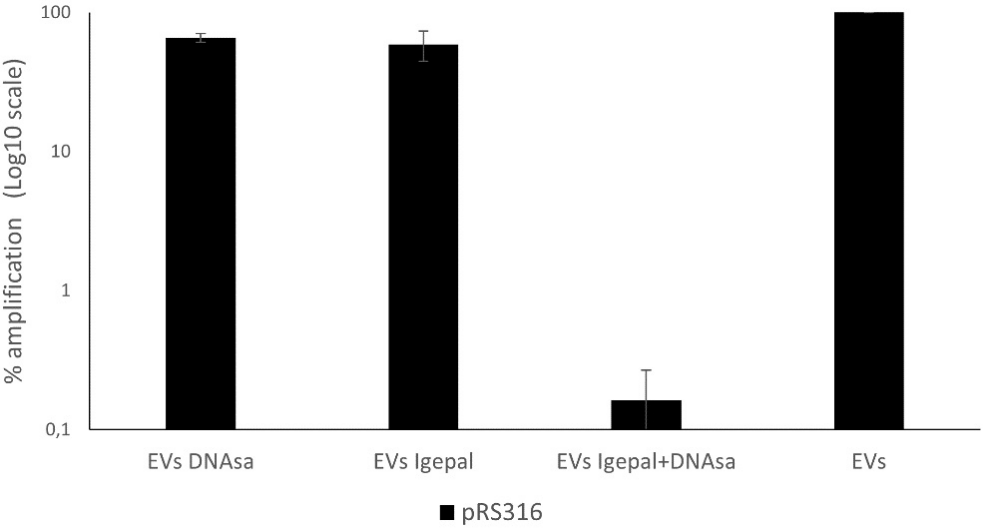
Remaining DNA in EV-enriched fractions from BY4741-pRS316 after detergent and DNase treatment. Four conditions were analyzed: untreated EVs, EVs treated with DNase, EVs treated with Igepal, and EVs treated with Igepal followed by DNase. DNA levels are expressed relative to the untreated EVs set as 100%. Data represent the mean ± standard deviation of three independent experiments. EV: Extracellular vesicle.

To determine whether the DNA detected in the EV-enriched fraction is genuinely encapsulated rather than derived from DNase-resistant structures caused by cell lysis or co-precipitation, DNase treatment was applied following membrane permeabilization with 0.5% Igepal. Membrane permeabilization made all DNA susceptible to degradation, confirming that the DNase-resistant fraction corresponds to DNA enclosed within the vesicles. These results indicate that the DNA detected in untreated EVs is protected inside the vesicles and not due to non-specific binding or external contamination [Figure 4].

### Transformation potential of pRS316-loaded EVs

To investigate the transformation potential of pRS316-loaded EVs, we incubated *S. cerevisiae* BY4743 strain with the EVs obtained from BY4741-pRS316 in minimal liquid medium without uridine to check if the correction of the auxotrophy through the incorporation of the plasmid was produced. We also plated the cells on agar plates without uridine. Additionally, we attempted to transform competent *E. coli* cells following a similar approach. Despite the presence of plasmid DNA in the EVs, no transfer of plasmid DNA to BY4743 yeast cells or *E. coli* was observed in either the liquid or solid media. In the liquid medium, there was no growth on selective media, and similarly, no colonies formed on agar plates, indicating that under the conditions tested, EV-mediated transfer was undetectable. 

### Plasmid origin affects DNA enrichment of EV fraction

To investigate how plasmid origin influences DNA incorporation into the EV-enriched fraction, we compared the presence of exogenous plasmid DNA and endogenous plasmid DNA (produced by *S. cerevisiae* strain BY4741 transformed with pRS316) in this fraction. Plasmids were introduced under two conditions: exogenous plasmid DNA added to a stationary-phase culture at a diluted concentration, and endogenous plasmid DNA synthesized by transformed yeast cells.

**Figure 5:**
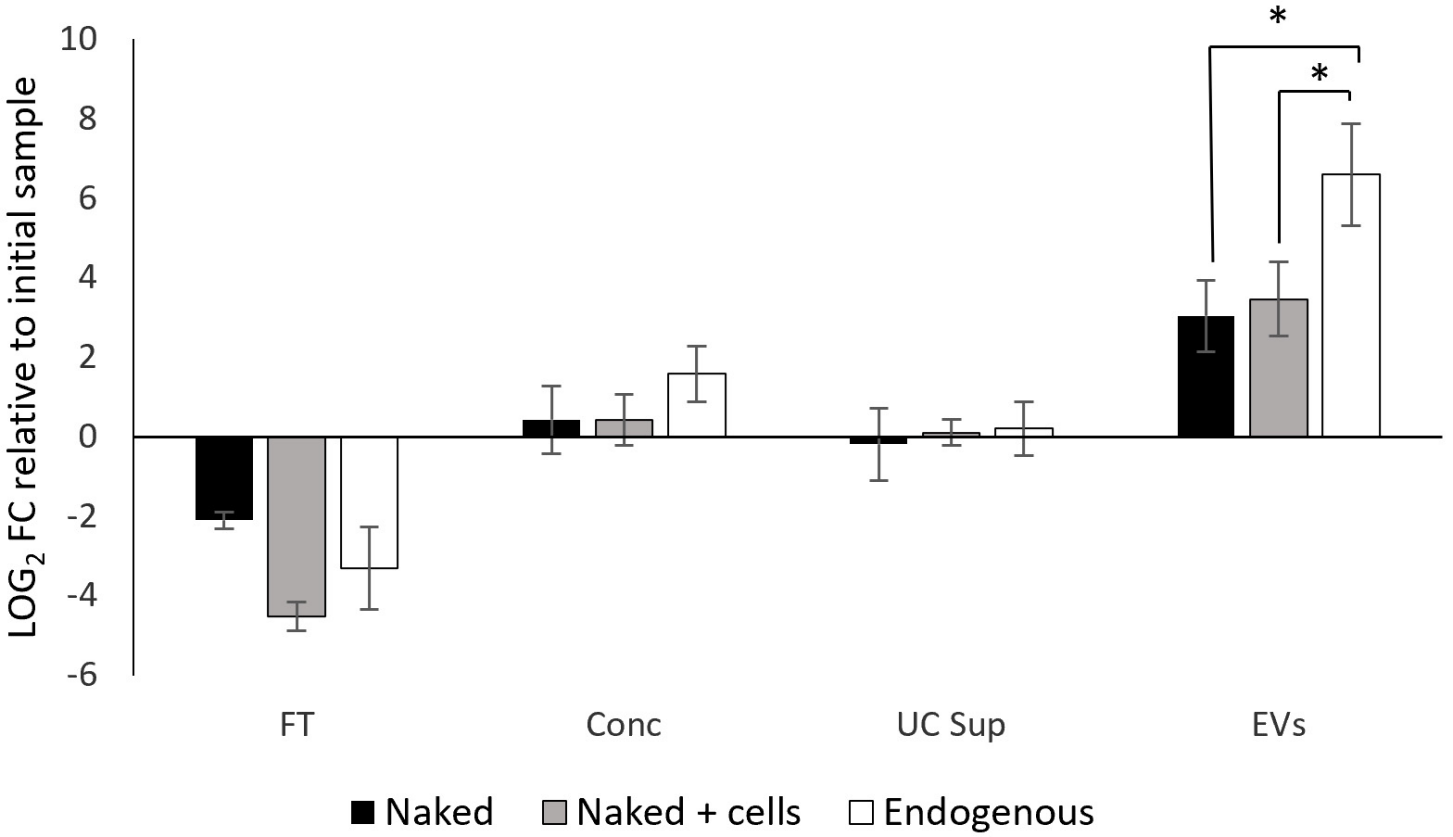
qPCR quantification (log_2_ FC) of pRS316 plasmid levels across fractions obtained during the EV isolation process, relative to the initial cell culture supernatant. The data are the means ± standard deviation of results from three independent experiments. Significant differences *vs.* EVs-enriched fraction of both naked plasmid conditions were determined by using the Student’s *t-*test (*, *P* < 0.05). Levels of endogenous plasmid were significantly higher compared to naked and naked + cells conditions. FT: Flowthrough; Conc: concentrated sample after tangential flow filtration; UC Sup: ultracentrifugation supernatant; EV: extracellular vesicle; qPCR: quantitative polymerase chain reaction; FC: fold change.

For the exogenous plasmid DNA, the concentration detected in the EV-enriched fraction showed an approximately 8-fold enrichment relative to the initial supernatant [Figure 5]. A control experiment performed under identical conditions but in the absence of yeast cells yielded similar results. Furthermore, the similarity between conditions with and without cells indicates that cell-derived factors do not contribute significantly to plasmid recovery in this context.

In contrast, when plasmid DNA was produced endogenously by yeast cells, a 97-fold enrichment in the EV fraction was detected. This marked difference suggests a distinct behavior between endogenous and exogenous plasmid DNA during EV isolation.

### Detection of ribosomal and mitochondrial DNA in EVs

To deepen the knowledge about the cargo carried by EVs and their role in cellular communication and gene transfer, we investigated the presence of other DNA molecules in the EVs, specifically ribosomal DNA (rDNA) and mitochondrial DNA (mtDNA). Gene coding for *COX1* and RDN18 were selected as markers. *RDN18* gene was chosen as a representative nuclear DNA element, as it is encoded by *RDN1* locus consisting of 100-200 repeated copies in tandem. This high copy number was expected to enhance the sensitivity of detection by qPCR.

**Figure 6:**
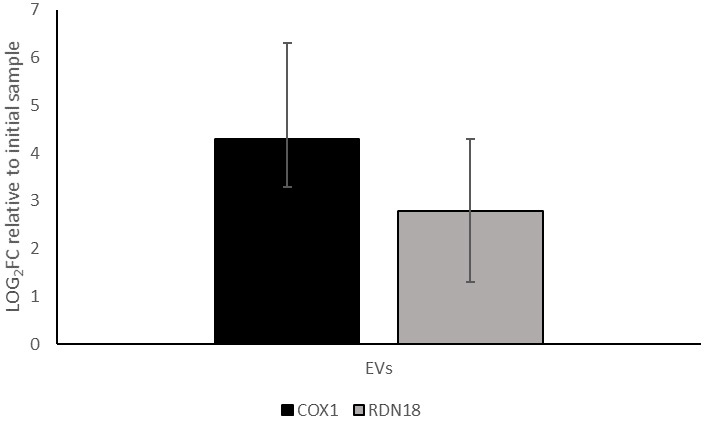
qPCR quantification (log_2_ FC) of *COX1* and *RDN18* expression in the EV-enriched fraction obtained from BY4741-pRS316, relative to their levels in the initial cell culture supernatant. Data represent the mean ± standard deviation from three independent experiments. *COX1*: Cytochrome c oxidase subunit I; *RDN18*: 18S ribosomal DNA; EV: Extracellular vesicle; qPCR: FC: fold change.

Consequently, we analyzed by qPCR the presence of these genes in the EVs released by BY4741-pRS316. The results showed that for *COX1*, the concentration in the EV fraction increased to an average of 19-fold enrichment relative to the initial supernatant, while for *RDN18* DNA it was concentrated approximately 7 times [Figure 6]. Thus, both rDNA and mtDNA were modestly concentrated in the EV fraction, albeit to a lesser extent compared to plasmid DNA whose concentration reached 97 times as mentioned above. 

In order to examine if the rDNA and mtDNA were encapsulated inside the vesicles, we treated the sample with DNase following the same procedure used in the previous experiments.

**Figure 7:**
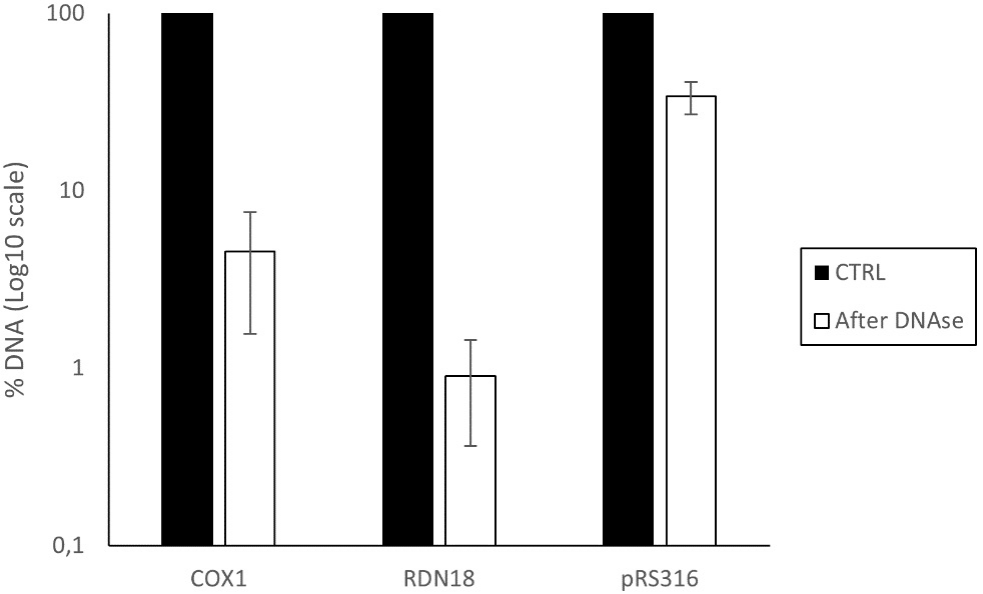
Percentage of DNA remaining after DNase treatment in the EV-enriched fraction isolated from BY4741-pRS316. The analysis includes mitochondrial DNA (*COX1*), ribosomal DNA (*RDN18*), and plasmid DNA (*pRS316*). Data represent the mean ± standard deviation from three independent experiments.* COX1*: cytochrome c oxidase subunit I; *RDN18*: 18S ribosomal DNA; EV: Extracellular vesicle; CTRL:

After DNase treatment, both mtDNA and rDNA showed only 4.5% and 0.9% remaining signal, respectively, compared to the untreated sample. This indicates that these DNA fragments were highly degraded, suggesting they were not efficiently encapsulated or protected within the EVs compared to the endogenous plasmid pRS316, at least for the sequences tested [Figure 7]. 

## DISCUSSION

EVs are known to carry nucleic acids, but the mechanisms governing DNA incorporation in yeast remain poorly understood. In this study, we investigated the presence and packaging of different types of DNA, focusing especially on plasmid DNA.

Our results demonstrate that plasmid DNA for pRS316 and YEp352 is consistently detectable in the EV-enriched fraction and becomes highly concentrated during the isolation process. This observation is in agreement with studies in mammalian systems reporting the presence of dsDNA in EVs^[28,29]^. However, PCR detection alone does not distinguish between DNA encapsulated inside vesicles and DNA merely associated with their surface.

DNase protection assays revealed distinct behaviors for the two plasmids studied. YEp352 was almost completely degraded, indicating that it remains accessible to DNase and is not effectively encapsulated within the EV membrane. In contrast, pRS316 exhibited partial resistance to DNase digestion. This protection is likely due to its encapsulation within the EV lipid bilayer, which may help shield the plasmid from enzymatic digestion. Lamichhane *et al*. reported that loading capacity of DNA in EVs depends on the length and structure of DNA, being linear molecules of less than 1,000 base pairs (bp) more efficiently incorporated than plasmids, so these findings align with a partial uptake of pRS316^[30]^.

Since both plasmids share similar characteristics, we hypothesize that the origin of replication may account for the observed differences in vesicle packaging and the resulting protection. Logically, we expected the high-copy-number plasmid to be encapsulated more efficiently than the low-copy-number plasmid. However, the results showed the opposite trend. It is well known that centromeric and 2 μm plasmids exhibit distinct features that affect segregation, copy number, stability, and replication mechanisms. Also, partitioning of the 2 μm plasmid involves plasmid-encoded DNA-binding proteins as well as host cell factors^[31]^, which could influence packaging. Indeed, previous studies have shown that when transcription was induced in hybrid CEN3-2 μm plasmids, plasmid copy number increased. However, the plasmids became highly unstable, emphasizing the functional differences between the two replication origins^[32]^. Also, it has been described that the replication origin influences DNA topology^[33,34]^. From our perspective, these factors highlight significant differences in the plasmid behavior, which could influence its incorporation into EVs.

Despite partial protection of pRS316 within EVs, no successful transformation of yeast or bacteria was achieved, indicating that under the conditions tested, pRS316-loaded EVs were not capable of mediating effective DNA transfer. This lack of transformation could be due to factors such as compromised plasmid integrity, the inability of EVs to efficiently deliver DNA into the target cells, or an insufficient concentration of plasmid in the sample. DNA carried within EVs from donor cells has been shown to have physiological relevance and to influence the function of recipient cells^[35]^. In bacteria, a positive outcome seems to depend on the plasmid properties and could be influenced by the experimental setup or even the vesicle origin. As an example, it has been reported that plasmid DNA-containing membrane vesicles from *Pseudomonas aeruginosa* were unable to transform the PAO1 strain or *E. coli* DH5α under a variety of conditions^[36]^. Nevertheless, successful plasmid transfer to *E. coli* mediated by EVs has also been observed, with evidence suggesting that factors such as plasmid size, EV dosage, and the origin of replication can influence the probability of vesicle-mediated transfer^[37]^.

Another finding of this work is that enrichment of the pRS316 plasmid in the EV-enriched fraction strongly depends on its origin. Exogenously added pRS316 DNA showed only slight enrichment relative to the initial supernatant, suggesting a largely passive process in which DNA partially co-sediments with EVs during isolation, likely due to centrifugal forces. The similarity between conditions with and without cells further indicates that cell-derived factors do not significantly contribute to this recovery.

In contrast, endogenously produced pRS316 in BY4741 cells was enriched nearly 100-fold in the EV fraction. These findings demonstrate that endogenously produced plasmid DNA is isolated far more efficiently than exogenously added DNA, suggesting that incorporation of the plasmid into the EV-enriched fraction is not merely a passive process. Instead, endogenous DNA likely benefits from active and selective packaging mechanisms, whereas exogenous DNA may associate with vesicles through non-specific, passive interactions or may be simply influenced by the experimental process. This observation supports recent studies indicating that cells may employ specific signals or protein-DNA interactions to direct particular DNA molecules into EVs^[38]^.

The analysis of mtDNA and rDNA further supports the selective nature of EV cargo loading. It has been reported that genomic DNA (gDNA) and other nuclear molecules can be transported by EVs as a mechanism for extracellular export. In cancerous cells, this content seems to reflect the genomic state of the cell^[39]^. Moreover, there is evidence of horizontal gene transfer through uptake of apoptotic bodies (a particular type of EVs) from tumour carrying gDNA, conferring advantages to the recipient cells^[40]^. Also, it has been shown that DNA within plasma-derived EVs is antigenically active and can bind to anti-DNA antibodies. This indicates that EV-associated gDNA may act as an extracellular signal, facilitating communication between apoptotic cells and immunological cells^[41]^.

Since rDNA is typically located in the nucleolus and is involved in the production of rRNA for the assembly of ribosomes^[42]^, its detection in EVs could be a reflection of the physiological status of the donor cell, or even point toward potential effects on receptor cells related to ribosomal function or biogenesis. Furthermore, it may act as an initial indication of the presence of other gDNA elements.

About mtDNA, it is essential for mitochondrial function and energy production, and it can escape into extracellular compartments under stress conditions^[43]^. The transfer of mtDNA between cells through EVs has demonstrated multiple biological effects, including energy support, signal dissemination, and the ability to trigger proinflammatory responses^[44]^. Thus, the presence of mtDNA in EVs of yeasts could potentially contribute to the coordination of responses to cellular damage or environmental stress.

The slight enrichment and high DNase sensitivity of mtDNA and rDNA in yeast EVs highlight the selective nature of cargo packaging. While mtDNA has been reported in EVs from mammalian cells^[21,45]^, studies examining its encapsulation in yeast EVs remain limited. In our analysis, mtDNA showed a moderate degree of association with EVs, but was still largely degraded by DNase.

In contrast, rDNA exhibited even lower persistence following DNase treatment, indicating a more transient or peripheral presence in the vesicle preparations. Notably, similar concentrations were observed in the extracellular EV fraction for the exogenously added plasmid (8-fold enrichment) and ribosomal RNA (7-fold enrichment). This observation suggests that passively sedimented DNA due to centrifugal forces could result in similar levels of concentration.

Although ribosomal RNA is consistently detected in fungal EVs^[23,46]^ our findings represent a first targeted assessment of rDNA in yeast EVs, revealing that it is neither efficiently encapsulated nor protected. These observations reinforce the idea that DNA incorporation into EVs is governed by selective, cargo-specific mechanisms and that not all DNA types are equally favored for packaging. Indeed, our results suggest that distinct types of plasmids are preferentially packaged, suggesting that plasmid incorporation may depend on specific plasmid properties.

Despite the insights provided by this study, several limitations should be acknowledged. First, our conclusions are based on the analysis of a limited number of plasmids, specifically pRS316 and YEp352; thus, the observations provide information but do not allow categorical conclusions. Although these plasmids mainly differ in their origin of replication, other uncharacterized features such as topology, stability, or interactions with host elements may also influence their incorporation into EVs. Therefore, attributing the observed differences in DNase protection solely to the replication origin should be interpreted with caution. Additional studies using a broader set of centromeric and 2 μm-based plasmids, as well as plasmids of varying size and structure, or strains naturally containing 2 μm plasmids, would be useful to strengthen these conclusions. Furthermore, although DNase treatment combined with membrane disruption supports the presence of plasmid DNA protected by the vesicular lipid bilayer, the limited amount of DNase-resistant DNA recovered from EVs restricts complementary analyses, including amplification of multiple plasmid regions by conventional PCR or other approaches that could help to determine whether the plasmid DNA is intact or fragmented. In addition, the EV isolation method based on differential centrifugation inevitably results in partial co-sedimentation of extravesicular DNA which cannot be completely eliminated and may contribute to the total DNA detected in EV-enriched fractions. Another limitation concerns the functional relevance of EV-associated plasmid DNA, as no successful transformation of yeast or bacterial recipient cells was achieved under the experimental conditions tested. This lack of transformation may reflect insufficient EV-associated DNA, compromised plasmid integrity, inefficient DNA delivery, or other experimental issues, rather than the absence of EV-mediated DNA transfer potential. In this point, it should be noted that, due to laboratory availability and the specific characteristics of the plasmid, only *E. coli* and the auxotrophic *S. cerevisiae* BY4743 strain were appropriate and therefore tested as recipient organisms. Consequently, the possibility that EV-mediated DNA transfer may occur in other recipients remains open. Finally, although mtDNA and rDNA were detected in EV preparations, their high sensitivity to DNase digestion limits conclusions regarding their relative abundance within EVs. Thus, additional gDNA targets could be evaluated in future analyses to complement these findings.

To date, limited information exists on DNA transport via EVs in yeasts, particularly regarding the mechanisms underlying DNA selection and protection. Our findings provide valuable new insights into the selective incorporation of different DNA types into yeast EVs, opening new avenues for research in microbial ecology and biotechnology. Further research is needed to elucidate the molecular basis of DNA packaging in yeast EVs and its functional significance.

## DECLARATIONS

### AI and AI-assisted tools statement

During the preparation of this manuscript, the AI tool ChatGPT (version GPT-4, released 2023-03-14) was used solely for language editing. The tool did not influence the study design, data collection, analysis, interpretation, or the scientific content of the work. All authors take full responsibility for the accuracy, integrity, and final content of the manuscript.

### Copyright

© The Author(s) 2026.
