# Supplementary material for: Selective DNA encapsulation in extracellular vesicles of Saccharomyces cerevisiae
Source: Extracell Vesicles Circ Nucl Acids. 2026 Mar 31;7(1):448–64. doi: 10.20517/evcna.2025.118 (PMC13074284; doi:10.20517/evcna.2025.118)
Supplement: Supplementary file 1 [file evcna-7-1-448-SupplementaryMaterials.pdf]

Selective DNA encapsulation in extracellular vesicles of *Saccharomyces cerevisiae*

Ana Perea-Martínez, Miguel Mejías-Ortiz, Pilar Morales, Ramon Gonzalez

Instituto de Ciencias de la Vid y del Vino (CSIC, Universidad de La Rioja, Gobierno de La Rioja), Logroño 26007, Spain.

Correspondence to: Dr. Ana Perea-Martínez, Instituto de Ciencias de la Vid y del Vino (CSIC, Universidad de La Rioja, Gobierno de la Rioja), Finca La Grajera, Carretera de Burgos, km 6, Logroño, La Rioja 26071, Spain. Email: ana.perea@icvv.es

ORCID: Ana Perea-Martínez (0000-0002-3300-0109)

Supplementary Table 1. List of primers used in this work

Region/Gene	Primer name	Primer sequence (5’-3’)	Application
2μ (replication origin)	YEp352 FW	AAGGAGCGAAAGGTGGATGG	PCR
2μ	YEp352 RV	ACCTCTGACACATGCAGCTC	PCR
CEN/ARS (replication origin)	pRS316 FW	GCCACCTGGGTCCTTTTCAT	PCR
CEN/ARS	pRS316 RV	CTTACACGCGCCTCGTATCT	PCR
Ampicillin resistance	qPCR AMP FW	CTACGATACGGGAGGGCTTA	qPCR
Ampicillin ressitance	qPCR AMP RV	CTGGATGGAGGCGGATAAAG	qPCR
COX1	qCOX1 FW	CTACAGATACAGCATTTCCAAGA	qPCR
COX1	qCOX1 RV	GTGCCTGAATAGATGATAATGGT	qPCR
RDN18	qRDN18 FW	AACTCACCAGGTCCAGACACAATAAGG	qPCR
RDN18	qRDN18 RV	AAGGTCTCGTTCGTTATCGCAATTAAGC	qPCR
